# Biological Activities and Bioavailability of Mangosteen Xanthones: A Critical Review of the Current Evidence

**DOI:** 10.3390/nu5083163

**Published:** 2013-08-13

**Authors:** Fabiola Gutierrez-Orozco, Mark L. Failla

**Affiliations:** 1 Interdisciplinary PhD Program in Nutrition, The Ohio State University, Columbus, OH 43210, USA; E-Mail: gutierrez-orozco.1@osu.edu; 2 Human Nutrition Program, The Ohio State University, Columbus, OH 43210, USA

**Keywords:** *Garcinia mangostana*, mangosteen, xanthones, α-mangostin

## Abstract

Mangosteen (*Garcinia mangostana* L.) is a tropical tree native to Southeast Asia that produces a fruit whose pericarp contains a family of tricyclic isoprenylated polyphenols referred to as xanthones. Numerous *in vitro* studies have shown that these xanthones possess anti-oxidant, anti-proliferative, pro-apoptotic, anti-inflammatory and anti-carcinogenic activities. Aggressive marketing of such health promoting benefits has resulted in mangosteen’s classification as a “superfruit”. This has led to sales of mangosteen containing beverages in USA alone exceeding $200 million in 2008 despite very limited animal and human studies. This review will (a) critically address recent reports of *in vivo* studies on the bioavailability and metabolism of mangosteen xanthones, (b) update the *in vitro* and *in vivo* data on anti-cancer and anti-inflammatory activities of mangosteen xanthones, and (c) suggest needed areas of inquiry regarding the absorption, metabolism and efficacy of mangosteen xanthones.

## 1. Introduction

Juice blends and other products containing exotic fruits, also known as *superfruits*, have been aggressively marketed for their proposed health benefits. This has resulted in a steady rise in sales of superfruit juices and products to consumers interested in their personal health. Mangosteen is one such superfruit that is produced by *Garcinia mangostana* L. The genus *Garcinia* is native to Asia and Africa and includes more than 300 distinct species from which several families of bioactive compounds such as xanthones, flavonoids, triterpenoids, and benzophenones have been isolated and characterized [1]. Although many *Garcinia* species including *G. mangostana*, *G. schomburgkiana*, *G. dulcis*, *G. cowa*, *G. atroviridis*, *G. hanburyi*, *G. bancana*, *G. xanthochymus*, *G. thorelii*, *G. hombroniana*, and *G. speciosa* bear edible fruits, mangosteen has captured the most attention in the market [2]. The mangosteen tree is mainly cultivated in Indonesia, Malaysia, the Philippines, and Thailand. Mature mangosteen trees range from 6 to 25 m. Production of the fruit generally requires 10 or more years with a yield of around 400 fruits per tree that is increased in older trees. Mangosteen fruit is round, dark purple or reddish, and has a white juicy pulp possessing a slightly acidic and sweet flavor that is enjoyed by many, and has resulted in it being referred to as the “queen of fruits”. The pericarp of mangosteen fruit has been used in traditional medicine in Southeast Asia for centuries to treat infection, wounds, inflammation and diarrhea [3].

Products containing mangosteen juice or extract are a fast growing segment of the functional beverages market. Aggressive marketing of the proposed health benefits of mangosteen has resulted in sales of mangosteen products in the US exceeding $200 million in 2008 [4]. Oftentimes, products marketed as mangosteen juice are a blend of numerous fruit juices with mangosteen being one of the less abundant components. For example, Xango^®^, one of the bestselling mangosteen products in the US, contains mangosteen puree, and a blend of juices from grape, pear, apple, blueberry, strawberry, raspberry, cranberry, and cherry.

**Figure 1:**
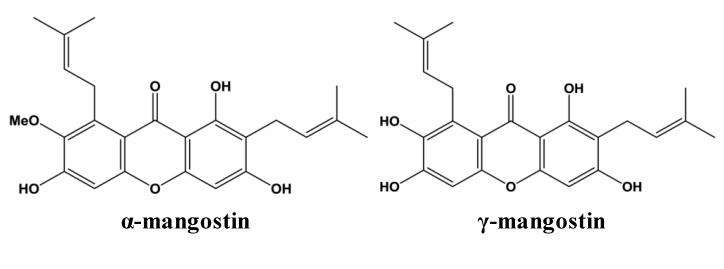
Chemical structures of two most abundant xanthones in mangosteen.

Secondary metabolites, known as xanthones, have been isolated from the pericarp of mangosteen and are attributed to the medicinal properties of the fruit. Xanthones have a unique chemical structure composed of a tricyclic aromatic system (C_6_–C_3_–C_6_). Isoprene, methoxyl and hydroxyl groups located at various locations on the A and B rings, resulting in a diverse array of xanthone compounds. Xanthones are found in a select few higher plant families. At least 68 distinct xanthones have been identified in different parts of the *G. mangostana* plant with 50 being present in the fruit’s pericarp at higher concentrations than in the aril or edible portion of the fruit [5]. The most abundant xanthones in the pericarp of mangosteen fruit are α- and γ-mangostin (Figure 1) [6]. Other xanthones in mangosteen pericarp include β-mangostin, gartanin, 8-deoxygartanin, garcinones A, B, C, D and E, mangostinone, 9-hydroxycalabaxanthone and isomangostin, among others. Details regarding the extraction and identification of these and other xanthones have been reviewed elsewhere [5].

**Figure 2:**
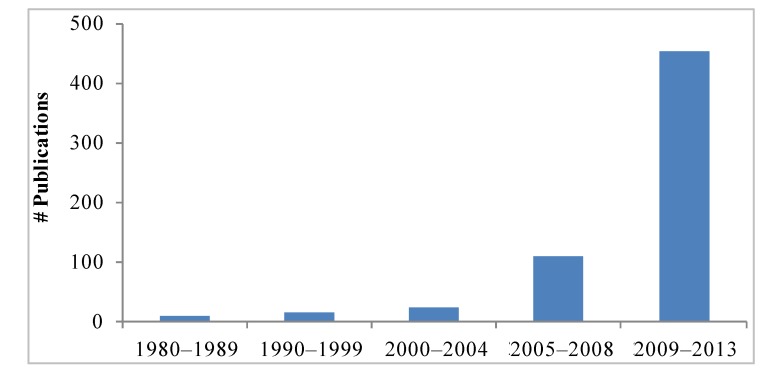
Number of publications related to mangosteen and their xanthones from 1980 to 2013. Search words: mangosteen, xanthones. Search performed on April 24 2013, including ahead of print publications. Databases: Pubmed, Science Direct, Google Scholar, Scirus.

Interest in the mangosteen fruit and xanthones has greatly increased in recent years as readily demonstrated by the number of scientific reports. A search of available literature using mangosteen and xanthones as terms in Pubmed, Science Direct, Google Scholar, and Scirus, retrieved 158 reports in the period of 1980–2008. In contrast, there have been 454 published articles from 2008 through March 2013 (Figure 2). By far, the most studied xanthone is α-mangostin (α-MG) for which anti-oxidant, anti-proliferative, pro-apoptotic, anti-inflammatory, anti-carcinogenic, and anti-microbial activities have been reported. Pertinent literature has been previously reviewed (Table 1). In this review, we focus primarily on recent reports considering the bioavailability and cellular metabolism of xanthones, their anti-cancer and anti-inflammatory activities, and their reported effects on cellular signaling pathways.

## 2. Metabolism and Bioavailability of Mangosteen Xanthones

**Figure 3:**
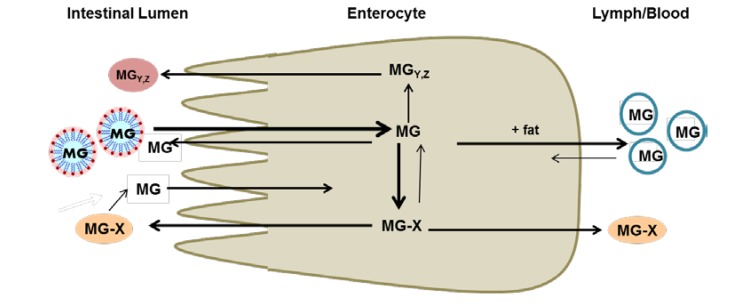
Transport and metabolism of mangosteen xanthones across intestinal epithelium. MG: xanthones; MG-X: xanthone phase II metabolites; MG_Y,Z_: bioconversion products of xanthones.

The first report on xanthone bioaccessibility and metabolism was performed using the coupled *in vitro* digestion/Caco-2 human intestinal cell model. Optimal bioaccessibility of α- and γ-MG xanthones was dependent on incorporation into bile salt mixed micelles. In addition, α-MG was transported across the apical surface of enterocyte-like Caco-2 cells and partially converted to phase II metabolites. Both unconjugated α-MG and its phase II metabolites were effluxed across the basolateral membrane suggesting that xanthones were absorbed. Transepithelial transport was enhanced by addition of products of lipid digestion in the apical compartment, suggesting that absorption was dependent on the assembly and secretion of chylomicrons. Xanthone metabolites also were retro-transported across the apical membrane into the simulated gut luminal compartment [7] (Figure 3). Recent studies in our laboratory have confirmed that human hepatic (HepG2), colonic (HT-29), enterocyte-like (Caco-2) and monocyte-like (THP-1) cell lines transport and metabolize α-MG to phase II conjugates. The extent of metabolism and degree of retention of α-MG and/or metabolites in the cell was dependent on the cell type. It is noteworthy that bioconversion of α-MG to other xanthones occurred in cultures of the cell lines, but not in primary cultures of human monocyte-derived macrophages. Among the xanthones identified in cultures were garcinone C (Caco-2 and HT-29 cells), garcinone D (THP-1 and HT-29 cells) and 9-hydroxycalabaxanthone (HepG2 and Caco-2 cells) [8].

**Table 1:** Available reviews on chemical properties and bioactivities of xanthones in mangosteen.

Chemical properties	Biological activities	Reference
natural and synthetic derivatives of xanthone	enzyme modulation, anti-tumor activity, anti-microbial, central nervous system(CNS) depressants, CNS stimulants, neurological disorders, anti-convulsant, analgesic, anti-arrhythmic, anti-hypertensive, anti-inflammatory, anti-allergic and immunomodulatory activities	[9]
xanthones isolated from pericarp, whole fruit, trunk, leaves and branches	anti-oxidant, anti-tumor, anti-inflammatory, anti-allergic, anti-bacterial, anti-fungal, anti-viral and anti-malarial activities	[3]
structural characterization of mangosteen xanthones in whole fruit, stem, aril, seeds, heartwood, leaves	anti-oxidant, anti-bacterial, anti-fungal, anti-malarial, anti-HIV, cytotoxic, aromatase inhibitory, anti-cancer and anti-inflammatory activities	[1]
chemical constituents and methods of isolation from pericarp, whole fruit, stem, aril, seeds, heartwood, leaves	anti-oxidant, anti-fungal, anti-bacterial, cytotoxic, anti-histamine, anti-HIV, CNS-depressant, cardiovascular, anti-inflammatory and anti-ulcerative activities	[5]
xanthones from mangosteen extracts	anti-cancer, anti-inflammatory, pro-apoptotic, cell cycle arresting, anti-invasive and anti-metastatic activities	[10]

The bioavailability (defined in this review as the fraction of an orally ingested or administered compound in a food, beverage or supplement reaching systemic circulation) and metabolism of α-MG have been reported in several recent studies with laboratory rodents. It was previously reported that intravenously injected α-MG (2 mg/kg) in rats was slowly eliminated from blood and rapidly distributed to tissues with a maximum concentration of 17.9 µg/mL. The bioavailability of orally administered α-MG (20 mg/kg dose) dissolved in an aqueous solution containing 2% ethanol and 2% Tween 80 was estimated as only 0.4% [11]. In a similar study, α-MG (40 mg/kg) dissolved in corn oil was orally administered to rats. The maximum plasma concentration (4.8 µg/mL) was reached within 63 min [12]. Xanthones were reported in plasma, liver and HT-29 subcutaneous tumors of athymic nude mice fed a diet containing 900 mg/kg α-MG (94% purity). Serum xanthones were extensively conjugated in these mice, whereas hepatic xanthones were primarily free. Interestingly, a xanthone tentatively identified as β-mangostin was the most abundant xanthone detected in liver, despite its very low abundance in the diet (85% *vs*. 0.3% of total xanthones, respectively). α-, β- and γ-Mangostin, 9-hydroxycalabaxanthone, 8-deoxygartanin, gartanin and garcinone E, were also detected in the HT-29 colon xenograft [13]. The presence of the xanthones in the tumor of mice fed diet with α-MG was associated with a 40% reduction in tumor mass of mice. The presence of high concentrations of α-MG (and metabolites) in feces of mice fed diet with α-MG suggests that the epithelium in the cecum and the colonic tissue is exposed to these xanthones. When α-MG was administered to C57BL/6 mice by oral gavage in an oil suspension (100 mg/kg), a maximum plasma concentration of 1.38 µmol/L was reached within 30 min. In addition, mono- and di-glucuronide metabolites of α-MG were detected in plasma. α-MG was detected in plasma 24 h after oral administration suggesting a slow elimination pattern [14].

There have also been several reports addressing the bioavailability of xanthones in human subjects. Healthy subjects consumed 59 mL of a xanthone-rich mangosteen juice product containing 94.2 mg xanthones. The maximum plasma concentration of α-MG (3.12 ± 1.47 ng/mL) was reached within 1 h. This study was limited by the fact that plasma samples were only collected for 6 h after ingestion of the mangosteen product and xanthone metabolites were not considered in the analysis. Plasma antioxidant capacity as measured by the oxygen radical absorbance capacity, ORAC, in these subjects was increased by as much as 18% after ingestion of the mangosteen product compared to subjects ingesting a placebo product. However, the contribution of α-MG to this increase in ORAC value is unknown since the beverage also contained green tea, aloe vera, and supplements including minerals, and vitamins A, B, C, D and E [15].

In a more recent human study, xanthones from 100% mangosteen juice (containing both liquid and pericarp particles) were found to be absorbed and partially conjugated by healthy adults ingesting a single dose (60 mL) of the mangosteen juice (containing 130 mg of xanthones) with a high fat Western-style breakfast. Both free and glucuronidated/sulfated xanthones (α- and γ-MG, garcinones D and E, 8-deoxygatanin and gartanin) were detected in serum and urine. Variability in maximum concentration of α-MG in serum (113 ± 107 nmol/L), as well as in time to maximum concentration (3.7 ± 2.4 h), was noted for the 10 subjects. Urinary excretion of xanthones accounted for 2% of the ingested dose [16]. Xanthones were still present in plasma 24 h after juice ingestion suggesting slow turnover as reported for mice after oral administration [14].

## 3. Anti-Cancer Activities of Xanthones

*In vivo* studies examining the anti-tumorigenic activities and *in vitro* anti-proliferative and pro-apoptotic activities of mangosteen xanthones with cancer cell lines are summarized in Table 2 and Table 3. The effects of mangosteen xanthones on mammary cancer have been examined in two studies using mammary BJMC3879 cancer cells xenografted into Balb/c mice. Subcutaneous α-MG [17] and dietary Panaxanthone (75%–85% α-MG and 5%–15% γ-MG) [18] significantly suppressed tumor volumes and metastastic expansion in this cancer model. *In vitro*, α-MG induced apoptosis, cell cycle arrest, activation of caspases-3 and -9, cytochrome c release and the loss of mitochondrial potential in BJMC3879 cells [17,18].

Two recent reports addressed the anti-tumorigenic effects of α-MG in glioblastoma and prostate xenograft mouse models. Intraperitoneal treatment with α-MG inhibited tumor growth by 50% in a GBM8401 glioblastoma xenograft model and this effect was associated with increased phosphorylation of AMPK (AMP-activated protein kinase) and induction of autophagy [19]. Oral administration of α-MG to athymic mice bearing 22Rv1 prostate tumors five times a week following cancer cell implantation significantly decreased tumor volume. *In vitro*, α-MG induced cell cycle arrest and apoptosis in 22Rv1 prostate cancer cells through activation of caspase-3. By using a cell free assay, α-MG also was shown to inhibit cyclin/cyclin-dependent kinase 4, which is involved in cell cycle progression [20].

The majority of *in vivo* studies examining the anti-cancer activity of mangosteen xanthones have focused on colon cancer. Dietary administration of α-MG significantly inhibited the induction and development of aberrant crypt foci (ACF) in a chemically-induced rat model of colon carcinogenesis. Less dysplasia, fewer lesions and decreased cell proliferation were also detected in α-MG-treated rats [21]. The growth of COLO 205 xenografts was completely suppressed when mice were injected intratumorally with 3 mg of a mangosteen extract containing α- and γ-MG. Caspase-mediated apoptosis was detected in the tumor cells. Lower doses of the extract also reduced tumor volume. Induction of COLO 205 cell apoptosis was also confirmed *in vitro* [22]. Dietary administration of an extract from mangosteen pericarp containing α- and γ-MG inhibited the growth of colorectal HCT116 xenografts in mice. *In vitro*, α-MG reduced HCT116 cell viability and induced caspase activation and loss of mitochondrial potential. In addition, mitogen-activated protein kinase/extracellular signal-regulated kinase (MAPK/ERK), Myc/Max and p53 signaling was enhanced by 71%, 48% and 30%, respectively, after treatment of cells with α-MG. An increase in Jun *N*-terminal kinase (JNK) pathway was observed, although the change failed to achieve statistical significance. Nuclear factor kappa-B (NF-κB) activity also was reduced by 30% [23]. Balb/c mice bearing colon cancer NL-17 xenografts showed 50%–70% reduction in tumor size when intraperitoneally treated with an extract from mangosteen pericarp containing 25% α-MG. Anti-proliferative activity of the extract on NL-17 cells was also confirmed *in vitro* [24]. Oral administration of α-MG also reduced growth of colon cancer Her2/CT26 xenografts in mice. The anti-tumor effect of α-MG was ascribed to autophagic activation rather than induction of endoplasmic reticulum stress as the xanthone was found to activate autophagy in the small intestine [25]. Finally, dietary α-MG reduced tumor mass of colon cancer HT-29 xenografts. In this study, xanthones and their metabolites were detected in serum, tumor, liver, and feces of these mice. *In vitro* analysis confirmed that α-MG inhibited HT-29 proliferation and decreased BcL-2 and β-catenin expression [13].

**Table 2:** *In vivo* anti-tumorigenic activities of mangosteen xanthones.

Cancer cell type	Animal model	Tested compound	Delivery route	Dose	Outcomes	Reference
BJMC3879 (murine mammary adenocarcinoma)	Balb/c	panaxanthone (75%–85% α-MG, 5%–15% γ-MG)	diet	5000 ppm	suppression of tumor volume and lung metastasis; decreased microvessel density	[18]
BJMC3879 (murine mammary adenocarcinoma)	Balb/c	α-MG	subcutaneous	20 mg/kg/day	decreased tumor growth and metastatic expansion; increased apoptosis; activation of caspase-3; decreased microvessel density; cytochrome c release from mitochondria; cell cycle arrest	[17]
GBM8401 (human malignant glioblastoma)	nude Balb/cA-ν (ν/ν)	α-MG	intraperitoneal	2 mg/kg/day	inhibition of tumor growth by 50%; increased phosphorylation of AMPK; induction of autophagy	[19]
22Rv1 (human prostate carcinoma)	Athymic nu/nu mice	α-MG	oral gavage	100 mg/kg 5x/week	decreased tumor growth	[20]
COLO205 (human colorectal adenocarcinoma)	Athymic NCr nu/nu mice	mangosteen pericarp extract containing 48 mg α-MG and 6.4 mg γ-MG per gram of extract	intratumorally	0.024–3.0 mg per tumor	complete suppression of tumor growth at 3 mg extract/tumor; apoptotic cells, nuclear fragmentation and chromatin condensation; activation of caspases-3 and -8	[22]
HCT116 (human colorectal carcinoma)	Athymic NCR nu/nu nude mice	extract of mangosteen pericarp (81% α-MG and 16% γ-MG)	diet	0.25% and 0.5% extract: food ratio (wt/wt)	inhibition of tumor growth; fewer blood vessels in tumor	[23]
NL-17 (murine colon adenocarcinoma)	Balb/c	pericarp methanolic extract (25% α-MG)	intraperitoneal	100–200 mg/kg	reduced tumor mass by 50%–70%	[24]
Her2/CT26 cells (murine colon carcinoma)	Balb/c	α-MG	oral	20 mg/kg	reduced subcutaneous growth	[25]
HT-29 (human colon adenocarcinoma)	Athymic Balb/c	α-MG	diet	900 mg/kg	40% reduction in tumor mass; decreased Bcl-2 and β-catenin	[13]
**Chemically induced cancer model**						
Chemically-induced (1,2dimethylhydra-zine) colon cancer	F344 rats	α-MG	diet	0.02% and 0.05% in CE-2 basal diet	inhibition of induction and development of ACF; decreased dysplastic foci and β-catenin accumulated crypts; lower proliferating cell nuclear antigen in colon	[21]

**Table 3:** *In vitro* pro-apoptotic and anti-proliferative activities of mangosteen xanthones.

Cell type	Tested compound	Dose	Outcomes	Reference
BJMC3879 (murine mammary adenocarcinoma)	α-MG	8 µM	induction of apoptosis; cell cycle arrest; activation of caspase-3 and -9; loss of mitochondrial potential	[18]
PC3, and 22Rv1 (human prostate carcinoma)	α-MG	2.5–15 µM	suppressed cell viability and colony formation; cell cycle arrest; activation of caspase-3	[20]
COLO205 (human colorectal adenocarcinoma)	mangosteen extract: 48 mg α-MG and 6.40 mg γ-MG/g extract	30 µg/mL	induction of apoptosis; activation of caspase-3 and -8; release of mitochondrial cytochrome c	[22]
HCT116 (human colorectal carcinoma)	extract of mangosteen pericarp (81% α-MG and 16% γ-MG)	10–20 µg/mL	reduced cell viability; increased activities of caspase-3/7 and-9; loss of mitochondrial potential; enhanced activity of MAPK/ERK, Myc/Max and p53 signaling; increased JNK; decreased NF-κB	[23]
NL-17 (murine colon adenocarcinoma)	pericarp methanol extract (25% α-MG)	>25 µg/mL	anti-proliferative activity	[24]
HT-29 (human colon adenocarcinoma)	α-MG	6–12 µM	anti-proliferative activity; decreased Bcl2 and β-catenin	[13]

## 4. Anti-Inflammatory Activity of Xanthones

The reported *in vitro* anti-inflammatory activities of mangosteen xanthones are summarized in Table 4. α-MG attenuated lipopolysaccharide (LPS)-induced expression of inflammatory mediators such as tumor necrosis factor α (TNF-α) and interleukin (IL-) 6 in human U937macrophage-like cells. α-MG also decreased activation of several signaling pathways including IL-1, mitogen-activated protein kinase kinase (MEK), JNK, ERK, signal transducer and activator of transcription 1 (STAT-1), and activator protein 1 (AP-1) in these cells [26,27]. Concentrations of α-MG used in these studies ranged from 6 to 12 nM [27] to 10–30 µM [26] and the LPS insult also differed in these reports. Inhibition of activation of MAPK, NF-κB, and AP-1 and attenuation of expression of pro-inflammatory cytokine genes also was observed in LPS-stimulated primary human adipocytes in response to α-MG treatment [7,28].

We recently examined the inhibitory effects of α-MG on the secretion of pro-inflammatory mediators by transformed and primary human cells. α-MG inhibited the secretion of IL-8 or TNF-α by human cell lines from various tissue origins challenged with a pro-inflammatory insult. Surprisingly, α-MG further stimulated the basal and LPS-stimulated secretion of TNF-α in primary cultures of human monocyte-derived macrophages cells [8].

α- and γ-MG inhibited nitric oxide (NO) and prostaglandin E_2_ (PGE_2_) production in murine RAW 264.7 macrophages. These effects were associated with reduced amounts of iNOS inducible NO synthase (iNOS) and cyclooxygenase-2 (COX-2) mRNA [29,30]. Suppression of histamine release by α-, β- and γ-MG was observed in IgE-sensitized rat basophilic leukemia RBL-2H3 cells [31]. γ-MG also dose dependently inhibited basal and A23187-induced release of PGE_2_ in C6 rat glioma cells [32]. These effects were associated with reduced COX-2 mRNA and protein expression, and NF-κB activation. Garcinone B also had similar effects in C6 cells by interfering with activation of NF-κB [33]. Contrary to these reports, an aqueous extract containing polyphenolic compounds from mangosteen pericarp stimulated the inflammatory response in cultures of Caco-2 cells treated with IL-1β [34]. This difference may be due to the absence of the hydrophobic xanthones in the extract.

The *in vivo* anti-inflammatory activities of mangosteen xanthones are summarized in Table 5. Early studies showed that both intraperitoneal and oral administration of α-MG, 1-isomangostin, or mangostin triacetate had anti-inflammatory activities in several rat models of inflammation [35]. The *in vivo* anti-inflammatory activity of γ-MG has been confirmed in the carrageenan-induced hind paw edema model in rats when the xanthone was administered intraperitoneally 30 minutes prior to inflammatory insult [36]. α-MG exhibited similar anti-inflammatory effects with this model [37]. Oral administration of α-MG also inhibited paw edema formation in mice [30]. Orally administered α- and γ-MG also exhibited anti-inflammatory activity in a mouse model of ovalbumin (OVA)-induced allergic asthma. Both xanthones had similar efficacy [38].

Information about the anti-inflammatory activity of mangosteen xanthones in humans is limited to three reports. Topical application of a gel containing extract of mangosteen pericarp decreased periodontal inflammation suggesting that the formulation may be useful as an adjuvant. However, the xanthone content and composition in the gel was not reported for this study [39]. Ingestion of a blended mangosteen juice decreased serum C-reactive protein (CRP) levels. However, other markers of inflammation were increased in subjects consuming the mangosteen product compared to placebo [40]. It was also reported that CRP levels in obese subjects consuming 18 oz of a mangosteen juice blend per day for 8 weeks were lower than those in the placebo group. However, levels of the pro-inflammatory interferon-inducible protein 10 (IP-10) and macrophage inflammatory protein-1 β (MIP-1 β) were increased in subjects consuming the high volumes of mangosteen juice blend [41].

## 5. Modulation of Pro-Apoptotic, Anti-Proliferative and Anti-Metastatic Signaling Pathways by Xanthones

A series of reports focused on the mechanisms of anti-proliferative and pro-apoptotic activities of xanthones in cultured cells have appeared recently and are summarized in Table 6. Mangosteen xanthones have been shown to mediate their pro-apoptotic effects by activating caspase cascade signaling in various cell types. Furthermore, mangosteen xanthones have been shown to disrupt mitochondrial membrane potential and release of cytochrome c from mitochondria into the cytoplasm. Less evidence and somewhat controversial findings have been reported on the effects of xanthones in other signaling pathways such as ERK1/2 and JNK1/2 with stimulation or inhibition of their activation, depending on cell type. α-MG was also shown to downregulate the levels of *p*-Akt, a protein kinase associated with cell survival. The anti-proliferative activity of α-MG in colorectal cancer cells were explained by inhibition of TCF/β-catenin transcriptional activity by the xanthone. Less is known about the effects of mangosteen xanthones on the cell cycle, although arrest at the G1 phase and downregulation of cyclins have been demonstrated in several studies.

The anti-metastatic potential of mangosteen xanthones was shown to be mediated by the inhibition of matrix metalloproteinase (MMP) activities which is expected to result in less adhesion, invasion and migration of cancer cells treated with α-MG. This suppressive effect was associated with an inhibition of IκBα degradation, as well as activation of the ανβ3 integrin/FAK/ERK pathway which is one of the main upstream regulators of NF-κB that inhibits its nuclear translocation.

**Table 4:** *In vitro* anti-inflammatory activities of mangosteen xanthone.

Cell type	Pro-inflammatory insult	Tested compound	Dose	Outcomes	Reference
Human U397 macrophage-likecells and primary adipocytes	LPS (100 µg/L) for 3 h	α- and γ-MG	α and γ-MG (2 h-pretreatment) with 10 or 30 µmol/L	α- and γ-MG decreased expression of IL-6, TNF-α, IFN-γ-inducible protein (IP)-10 in macrophage-like cells; decreased phosphorylation of MEK, JNK, ERK and p38; only γ-MG pretreatment attenuated LPS-mediated IκBα degradation; α- and γ-MG pretreatment decreased phosphorylation of c-Jun, Elk-1 and ATF-2; α- and γ-MG attenuated LPS-induced PPAR-γ suppression; γ-MG reduced inflammation and insulin resistance in adipocytes	[26]
Human primary adipocytes	LPS,10 µg/L for 3 h	α-MG and γ-MG	α- or γ-MG (24 h pretreatment with 3 µmol/L)	α- and γ-MG attenuated LPS-induced inflammatory gene expression of TNF-α, IL-1β, IL-6, IL-8, MCP-1, and Toll-like receptor-2; α- and γ-MG decreased MAPK activation by suppressing phosphorylation of JNK, p38, and ERK; γ-MG attenuated IκBα degradation and NF-κB activation induced by LPS; xanthones inhibited phosphorylation of c-jun and transcriptional activity of AP-1; γ-MG blocked LPS-induced suppression of PPARγ (peroxisome proliferator-activated receptor γ) and its target genes	[28]
Human U397 macrophage-like cells	LPS (0.1 ng/mL) for 4 h	α-MG	6–12 nM for 30 min	α-MG attenuated LPS-stimulated TNF-α secretion by U937 macrophage-like cells and suppressed expression of genes related to immune responses and inflammatory processes such as cytokine production, Th1 and Th2 differentiation, and IL-1 signaling; α-MG decreased activation of p38, ERK1/2, JNK, STAT1, c-Fos and c-Jun	[27]
Human cells: primary monocyte-derived macrophages (MDM); macrophage-like THP-1, hepatic HepG2, enterocyte-like Caco-2, and HT-29 colon adenocarcinoma	LPS (100 ng/mL for MDM and HT-29, 0.1 ng/mL for THP-1); PMA (50 ng/mL for HepG2); IL-1β (5 ng/mL for Caco-2)	α-MG	4.5–10 µM (pretreatment for various times)	inhibition of IL-8 secretion by Caco-2, HT-29 and THP-1 cells; inhibition of TNF-α by HepG2 cells; stimulation of TNF-α by primary MDM cells	[8]
Murine RAW 264.7 macrophage-like	LPS (100 µg/mL)	pericarp ethanol extract, α- and γ-MG	pericarp ethanol extract, (3–100 µg/mL), α- and γ-MG (3–100 µM)	α-MG and γ-MG inhibited NO and PGE_2_ production with moderate inhibitory effects on secretion of TNF-α and IL-4; expression of iNOS and COX-2 mRNA suppressed by α-MG; γ-MG inhibited transcription of iNOS	[29]
Murine RAW 264.7 macrophage-like	LPS (0.5–1 µg/mL)	α- and γ-MG	3–25 µM	inhibition of NO and PGE_2_ production by α- and γ-MG; iNOS expression reduced by both compounds; COX-2 expression and iNOS enzymatic activity were not affected	[30]
Rat RBL-2H3 basophilic leukemia	bovine serum albumin	α-, β-, and γ-MG	20 µM	α-MG significantly inhibited histamine release and blocked cytoplasmic Ca^2+^ elevation; γ-MG significantly reduced reactive oxygen species; suppressed phosphorylation of Syk, phospholipase C γ1 and γ2 by all mangostins; complete suppression of phosphorylation of Erk ½; JNK ½ and p38 MAPK signaling not altered; slight suppression of *p*-Akt; decreased phosphorylation of ERK and cytosolic phospholipase A_2_	[31]
Rat C6 glioma cells	A23187 calcium ionophore (10 µM)	γ-MG	1–30 µM	inhibition of COX-1 and -2 activities and PGE_2_ release by γ-MG; no effects on MAPK/ERK phosphorylation	[32]
Rat C6 glioma cells	LPS (10 µg/mL)	γ-MG	1–30 µM	inhibition of LPS-induced PGE_2_ release, COX-2 mRNA and protein expression; no effect on COX-1; inhibition of IκB kinase activity; inhibition of IκB degradation; decreased NF-κB activation	[36]
Rat C6 glioma cells	A23187 calcium ionophore (10 µM) and LPS (1 µg/mL)	Garcinone B	10–20 µM	inhibition of COX-1 and COX-2 activities and PGE_2_ release; inhibition of IKK activity and NF-κB-dependent transcription	[33]
Human Caco-2 enterocyte-like cells	IL-1β (25 µg/mL)	aqueous extract of mangosteen pericarp containing polyphenolic compounds	50 µmol gallic acid equivalents/L	stimulation of basal PGE_2_ secretion; no effect on IL-8 secretion or activation of ERK, JNK, and NF-κB	[34]

**Table 5:** *In vivo* anti-inflammatory activities of mangosteen xanthones.

Animal studies					
Model	Tested compound/product	Delivery route	Dose	Outcomes	Reference
rats: carrageenan-induced hind paw edema, cotton pellet implantation, granuloma pouch technique	α-MG, 1-isomangostin, mangostin triacetate	intra-peritoneal, oral	50 mg/kg	reduction in paw edema volume, granuloma weight, and granuloma pouch exudate	[35]
rat carrageenan-induced hind paw edema	γ-MG	intra-peritoneal	10 and 30 mg/kg	concentration dependent inhibition of edema formation	[36]
rat carrageenan-induced paw edema	α-MG isolated from *Allanblackiamonticola*	not specified	9.4 mg/kg	inhibition of edema	[37]
mouse carrageenan-induced paw edema	α- and γ-MG	oral	20 mg/kg	inhibition of paw edema formation by α-MG, but not by γ-MG	[30]
mouse OVA-induced allergic asthma	α- and γ-MG	oral	10 and 30 mg/kg	both xanthones attenuated inflammatory cell recruitment into the airway; reduced airway hyper-responsiveness; lower levels of Th2 cytokines; attenuated PI3K activity, Akt phosphorylation, and NF-κB activation	[38]
**Human studies**					
human subjects with periodontal pockets	pericarp extract	topical	not specified	clinical improvement in periodontal inflammation; subgingival microbial composition altered from diseased to healthy state	[39]
healthy adults	mangosteen supplement containing mangosteen juice, vitamins, minerals, aloe vera, and green tea	oral	59 mL/day for 30 days	decreased levels of serum CRP levels; increased ratio of T helper to cytotoxic T cells; elevated serum levels of IL-1α and IL-1β, and complement components C3 and C4	[40]
obese subjects	mangosteen juice blend (mangosteen, apple, pear, grape, blueberry, raspberry, strawberry, cranberry and cherry)	oral	6, 12, and 18 oz/day for 8 weeks	Decreased CRP levels in subjects consuming 18 oz of blended juice; increased levels of IP-10 in subjects consuming 6 and 18 oz of blended juice; no differences in F2 isoprostane and IL-12p70 levels; increased MIP-1 beta in subjects ingesting 18 oz blended juice	[41]

**Table 6:** Modulation of signaling pathways related to apoptosis, cell cycle and metastasis by mangosteen xanthones.

Biological activity	Target/messenger/process	Cell type	Reference
**Apoptosis**	*↓p-ERK1/2; ↓p-JNK1/2	chondrosarcoma SW1353	[42]
	↓Ψ_m_	leukemia HL60; prostate cancer PC12; colorectal cancer DLD-1; melanoma SK-MEL-28; colorectal HCT116; malignant glioblastoma GBM 8401	[23,43,44,45,46,47]
	^¥^↑Caspase-3	chondrosarcoma SW1353; colon cancer COLO205; leukemia HL60; prostate cancer PC12; melanoma SK-MEL-28; colorectal HCT116; breast cancer MDA-MB231	[22,23,42,43,44,45,48]
	↑Caspase-8	chondrosarcoma SW1353; colon cancer COLO205; breast cancer MDA-MB231	[22,42,48]
	↓Bcl-2; ↑Bax	chondrosarcoma SW1353	[42]
	↑cytochrome c release	chondrosarcoma SW1353; colon cancer COLO205; leukemia HL60; prostate cancer PC12; breast cancer MDA-MB231	[22,42,43,44,48]
	↓Akt	chondrosarcoma SW1353; colorectal cancer DLD-1	[42,46]
	↑p-JNK1/2	prostate cancer PC12; colorectal cancer DLD-1	[44,46]
	↑Endonuclease G	colorectal cancer DLD-1	[46]
	↑p-ERK1/2	colorectal cancer DLD-1 and HCT116	[23,46]
	↑microRNA-143	colorectal cancer DLD-1	[46]
	↓NF-κB	colorectal HCT116	[23]
	↑Myc, Max, p53	colorectal HCT116	[23]
	↓β-catenin	colorectal HCT116 and SW480	[49]
**Cell cycle**	G1 arrest	colorectal cancer DLD-1; melanoma SK-MEL-28; breast cancer MDA-MB231	[45,48,50]
	↓cyclins, cdc2	colorectal cancer DLD-1; breast cancer MDA-MB231	[48,50]
**Metastasis**	↓MMP-2, MMP-9	prostate carcinoma PC-3; breast adenocarcinoma MCF-7; lung adenocarcinoma A549	[51,52,53]
	↓u-PA^+^	prostate carcinoma PC-3	[51]
	↓p-JNK1/2	prostate carcinoma PC-3	[51]
	↓NF-κB	prostate carcinoma PC-3; breast adenocarcinoma MCF-7; lung adenocarcinoma A549	[51,52,53]
	↓AP-1	prostate carcinoma PC-3; breast adenocarcinoma MCF-7	[51,52,53]
	↓p-ERK1/2	breast adenocarcinoma MCF-7; lung adenocarcinoma A549	[51,52,53]
	↓ανβ3 integrin/FAK	lung adenocarcinoma A549	[51,52,53]

*↓, decrease; ^¥^↑, increase; Ψ_m_, mitochondrial membrane potential; ^+^u-PA, urokinase-plasminogen activator.

## 6. Future Research

Two human studies have reported that ingestion of a mangosteen juice blend or a xanthone-rich mangosteen product decreased serum CRP levels and increased ORAC values, respectively [15,41]. However, increased levels of several pro-inflammatory mediators were also observed. Results from these studies should be considered with caution as there is no way to discriminate the effects of other components in these products. Although no adverse events were reported in these trials, the potential long term toxicity of products containing mangosteen xanthones requires assessment.

With the exception of our study on primary human monocyte-derived macrophages [8], the effect of mangosteen xanthones on cultured normal cells has not been addressed. Our preliminary data suggest that primary and transformed human cells respond differently to α-MG with the xanthone promoting the secretion of pro-inflammatory mediators by normal cells. It is expected that cell phenotype will affect the response to bioactive compounds but whether this difference is limited to the inflammatory response in human cells is not clear. α-MG has been shown to promote apoptosis in cancer cells but this activity has not been evaluated in cells with a non-cancerous phenotype. Differences between normal and cancer cells may also be explained by the degree of metabolism and types of metabolic products. Our results also showed that while transformed human cell lines convert α-MG to phase II metabolites and other xanthones, only phase II metabolites are detected in primary cell cultures.

The vast majority of studies have focused on the anti-cancer and anti-inflammatory activities of mangosteen xanthones, and particularly α-MG. Our work and that of others has shown that α-MG and other xanthones are metabolized by human and animal cells. Thus, the possibility of metabolites of xanthones exerting some of the observed effects cannot be ruled out. It has been reported that metabolites of various phytochemicals possess the observed bioactivities [54,55,56,57]. Further investigation is needed to elucidate whether individual metabolites and/or their combinations possess some of the bioactivities primarily attributed to mangosteen xanthones. Similarly, more pharmacokinetic studies are needed to assess the bioavailability of xanthones from mangosteen containing beverages and food products.

One aspect of *in vitro* studies that is often neglected is the stability of the compound in cell culture conditions. Many polyphenolic compounds readily react with components of cell culture media to generate H_2_O_2_, quinones and semiquinonescapable of inducing alterations in cellular activities [58,59]. Studies on our laboratory showed that α-MG was readily degraded in different types of serum-free media (*i.e.*, RPMI, DMEM, MEM and McCoy’s 5A) when added in a dimethyl sulfoxide (DMSO) stock solution [8]. We stabilized the xanthone by delivering DMSO-solubilized α-MG to media containing serum or by delivering the pure compound in Tween 40 micelles. Previous studies of the biological activities of α-MG have generally used DMSO as delivery vehicle [29,30], although it is unclear if serum was present during experiment.

Although the bioavailability of mangosteen xanthones is limited as it is for many phytochemicals, the gastrointestinal (GI) tract is exposed to high concentrations of these compounds and their metabolites. Consequently, we are currently examining the effects of dietary α-MG in a mouse model of ulcerative colitis. It is possible that the bioactivities of xanthones and/or metabolites may differ depending on their concentrations in biofluids and cells. If activities are mediated by inducing an adaptive stress or “hormetic” response [60,61], higher concentrations of these compounds (such as those found in the GI tract) may not necessarily be beneficial. Finally, consideration of the reported anti-microbial activity of xanthones on the gut microbiome merits consideration.

Despite the numerous *in vitro* and *in vivo* studies on the bioactivities of mangosteen xanthones, more research is needed to evaluate their safety and health benefits before they can be recommended for preventive or therapeutic purposes.

## 7. Conclusions

Various health-promoting activities of xanthones in the pericarp of mangosteen fruit have been described by numerous investigators using *in vitro* cellular models. During the past several years, anti-tumorigenic and anti-inflammatory activities of xanthones have been demonstrated in laboratory rodents. Controlled intervention trials of the efficacy of xanthones in human volunteers, as well as characterization of the absorption, metabolism and elimination of these compounds, remain quite limited. Also, the potential toxicity of chronic ingestion of formulations containing mangosteen pericarp and its extracts has received minimal attention. Despite the numerous health claims on advertising sites for producers and retailers of products and beverages containing mangosteen, there is insufficient scientific evidence at this time to support the use of mangosteen containing supplements as enhancers of health and useful adjuvants for treatment of various pathophysiological illnesses.
